# A Case of COVID-19 With Myasthenic Crisis

**DOI:** 10.7759/cureus.24936

**Published:** 2022-05-12

**Authors:** Chenfan Xia, Ernest Butler, Rachel Edwards, Navin Amarasinghe

**Affiliations:** 1 Department of Medicine, Frankston Hospital, Melbourne, AUS; 2 Department of Neurology, Frankston Hospital, Melbourne, AUS; 3 Department of Medicine, Casey Hospital, Melbourne, AUS

**Keywords:** covid-19 infection, abg, steroid use, myasthenia gravis treated with high dose steroid, covid-19, myasthenic exacerbation, myasthenia gravis (mg)

## Abstract

Coronavirus disease 2019 (COVID-19) infection can increase the risk of myasthenic crisis. Dexamethasone has been widely used to manage severe COVID-19 infection. Paradoxically, steroids are effective for treating myasthenia gravis; however, when they are started in high doses, there is an associated risk of steroid-induced exacerbation.

This case report describes an 86-year-old male with seropositive generalised myasthenia gravis, whose course had been stable for years. At the time of his COVID-19 diagnosis, he was on pyridostigmine and prednisolone 10 mg daily. He was treated with IV dexamethasone 6 mg daily, remdesivir, and antibiotics. On day 10 of admission, he had a sudden deterioration with a Glasgow Coma Scale (GCS) score of 3. Arterial blood gas (ABG) showed a new type 2 respiratory failure suggesting myasthenic crisis. Although his ABG improved after commencing bilevel positive airway pressure (BiPAP), his condition continued to deteriorate and he died the next day. A decision not to intubate and ventilate had been made given his poor clinical state and low chance of recovery.

His myasthenic crisis was likely precipitated by the COVID-19 infection, although steroids, azithromycin, and doxycycline also have the potential to cause the worsening of myasthenia gravis. Further studies are needed to evaluate the efficacy and risk of steroid use in this patient population. Ventilatory failure may occur insidiously and is often difficult to detect, especially in elderly and delirious patients in whom performing a neurological examination can be difficult. Regular ABG and bedside measures of forced vital capacity may be considered to monitor the development of type 2 respiratory failure.

## Introduction

Coronavirus disease 2019 (COVID-19) infection can lead to an increase in the risk of myasthenic crisis^ ^[1,2]. Dexamethasone has been extensively used to manage severe COVID-19 infection. Paradoxically, steroids can be effective for treating myasthenia gravis, yet when initiated in high doses, it is associated with the risk of steroid-induced exacerbation [3,4]. In this report, we present the case of a patient with seropositive generalised myasthenia gravis who unfortunately died from COVID-19 infection and myasthenic crisis after undergoing treatment with dexamethasone and remdesivir.

## Case presentation

An 86-year-old male was admitted to the hospital during the severe acute respiratory syndrome coronavirus 2 (SARS-CoV-2) Delta variant (B.1.617.2) pandemic in Australia for dyspnea, cough, fever, and lethargy on day six following a positive COVID-19 test, taken after a nosocomially acquired contact during admission at a different hospital for a stage 2-3 sacral pressure area that had been healing. He had been pre-morbidly living at home with his wife on a home-care package and subsequently moved to a nursing home for respite and isolation. He had a history of seropositive (positive acetylcholine receptor antibody) myasthenia gravis diagnosed in 2007. His course had been stable for years before previously being complicated by dysphagia and recurrent respiratory tract infections requiring nasogastric tube feeding and intravenous immunoglobulin (IVIG). At the time of his COVID-19 diagnosis, he was on pyridostigmine 60 mg four times a day, which was continued during the admission, and prednisolone 10 mg daily, which was stopped after commencing dexamethasone 6 mg daily on day two. He had no other significant medical history and had received a dose of the ChAdOx1 nCoV-19 vaccine around the time of exposure, two weeks prior to the positive test.

**Figure 1:**
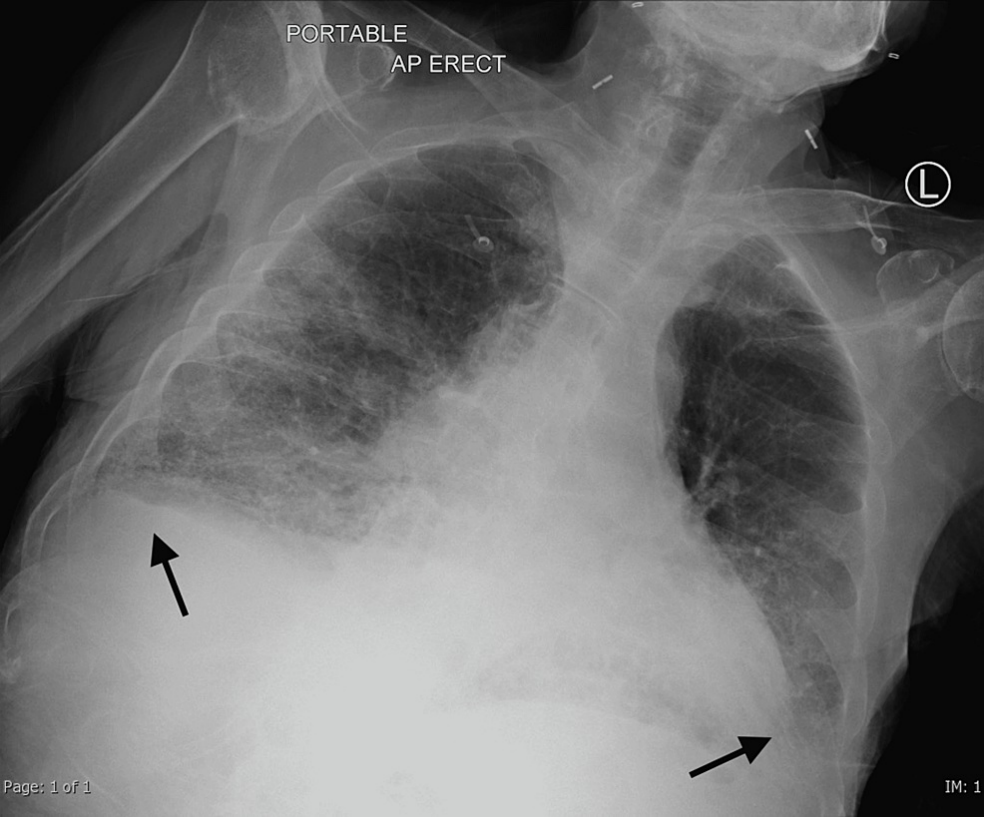
Chest X-ray showing extensive bilateral air space infiltration and small bilateral pleural effusions (arrows)

At presentation, he had mild tachypnea, oxygen saturation of 93% on room air, blood pressure of 93/57 mmHg, and a heart rate of 70 beats per minute. He had no ptosis, normal eye closure, no slurred speech, and no dysphagia. He was able to talk in full sentences and tolerate a regular diet. He had mild generalised weakness and shortness of breath on exertion, when rested in bed, and required a standing machine for transfer. His myasthenia gravis composite score was 7/50. Labs on admission were significant for elevated C-reactive protein (CRP) of 284 mg/L (reference range: 0-10 mg/L), procalcitonin of 4.87 ng/ml (reference range: <0.5 ng/ml), ferritin of 1525 ug/L (reference range: 30-320 ug/L), fibrinogen of 7.6 g/L (reference range: 1.5-4.0 g/L), d-dimer of 3.19 ug/ml (reference range: <0.5 ug/ml), and mild leucocytosis of 11.1 x 10^9^/L (reference range: 4-11 x 10^9^/L). Relevant normal results included a creatine kinase (CK) of 122 U/L (reference range: 0-240 U/L), lactate of 1.7 mmol/L (reference range: 0.5-2 mmol/L), and lactate dehydrogenase (LDH) of 203 U/L (reference range: 120-250 U/L). His chest X-ray showed extensive bilateral air space infiltration and small bilateral pleural effusions (Figure 1). He was started on antibiotics including ceftriaxone and azithromycin for two days, followed by ceftriaxone and doxycycline for five days, and then cefuroxime only.

On day two of admission, the patient became hypoxic with an oxygen saturation of 94% with 2 litres of nasal prongs. He was upgraded from his usual 10-mg of prednisolone to IV dexamethasone 6 mg daily, and five days of IV remdesivir with an initial dose of 200 mg followed by 100 mg daily. He showed some improvement after dexamethasone and remdesivir initially, and did not require supplemental oxygen for a day; however, he became hypoxic again, with increasing requirements of 4 litres of the nasal prongs.

**Figure 2:**
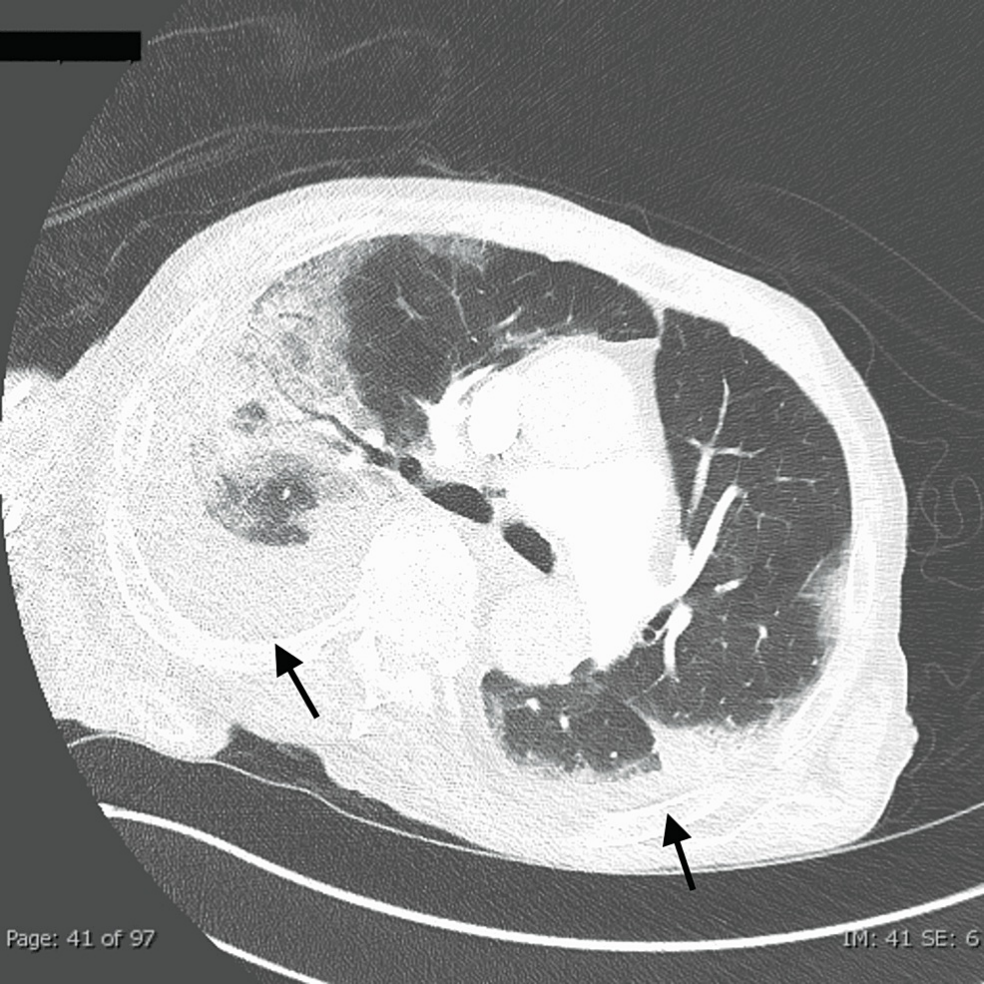
CTPA showing bilateral pulmonary parenchymal opacities and moderate bilateral pleural effusion (arrows) CTPA: computed tomography pulmonary angiogram

On day eight, a CT pulmonary angiogram showed bilateral pulmonary parenchymal opacities and moderate bilateral pleural effusions but no pulmonary embolism (Figure 2).

He was started on IV frusemide 40 mg BD. On day nine, his oxygen requirement increased further to an oxygen saturation of 90% on a 15-litre nonrebreather mask. His condition subsequently improved after a few hours back to 2 litres of nasal prongs with an oxygen saturation of 96%. He also had one dose of baricitinib.

On day 10, he had a sudden deterioration with a MET call for a Glasgow Coma Scale (GCS) score of 3 and was subsequently found to have hypoxia with an oxygen saturation of 78% with nasal prongs, which improved after bilevel positive airway pressure (BiPAP) but still had a reduced level of consciousness (GCS score of 11: eye-opening response of 4 + best verbal response of 2 + best motor response of 5). Arterial blood gas (ABG) showed PH of 7.11, PO_2_ of 76 mmHg, PCO_2_ of 93 mmHg, and HCO_3_ of 28 mmol/L compared with earlier ABG on day seven of admission (PH: 7.50, PO_2_: 67 mmHg, PCO_2_: 34 mmHg, HCO_3_: 26 mmol/L), suggesting a new type 2 respiratory failure.

Type 2 respiratory failure most likely occurred due to respiratory muscle weakness and myasthenic crisis. Pneumonia alone often leads to type 1 respiratory failure. Our patient had no other risk factors for type 2 respiratory failure, had no history of chronic obstructive pulmonary disease, and did not have any sedatives or opioids during his admission. However, he deteriorated quickly. We could not perform repetitive nerve stimulation or antibody titre to confirm the diagnosis of myasthenic crisis. Neurological examination was also challenging due to his reduced GCS score. Although his ABG improved after commencing BiPAP to PH of 7.49, PO_2_ of 61 mmHg, PCO_2_ of 32 mmHg, and HCO_3_ of 24 mmol/L, he, unfortunately, died the next day. A decision not to intubate and ventilate had been made given his poor clinical state and low chance of recovery.

## Discussion

Our patient had multiple risk factors for the myasthenic crisis, especially COVID-19 infection and high-dose steroids. He was on azithromycin and doxycycline as well. It is also uncertain whether the vaccine he had two weeks prior to the positive test would be relevant in any way.

In patients hospitalised with COVID-19 infection, dexamethasone 6 mg daily for up to 10 days is beneficial for lowering mortality rates among those receiving either oxygen alone or invasive mechanical ventilation [5]. There is limited data regarding steroid use in COVID-19 with myasthenia gravis. One case study reported a patient successfully weaned off a ventilator post-COVID-19 infection who had refractory myasthenia gravis for which he was on prednisolone 40 mg daily and treated with dexamethasone 6 mg and remdesivir [6]. Another study reported a case of myasthenia gravis, on prednisolone 30 mg daily, who developed a myasthenic crisis and was intubated and treated with plasma exchange and stress-dose steroid therapy with no additional specific COVID-19 treatment; the patient subsequently recovered [7]. Regarding mild COVID-19 disease, there has been a case report of a patient with refractory myasthenia gravis on multiple long-term immunosuppressive therapies including prednisolone 30 mg daily who recovered successfully with no changes to her immunosuppressive therapy [8]. Azithromycin has been studied as an adjunctive treatment for patients with COVID-19, with apparently favourable results. However, one study suggests that it is not recommended in myasthenia gravis without available ventilatory support [9].

Ventilatory failure may occur suddenly or insidiously and is often difficult to detect [10], especially in elderly and delirious patients in whom performing a neurological examination and assessing respiratory muscle function can be difficult. Although abnormalities of arterial blood gases are insensitive measures of respiratory muscle weakness [10], regular ABG and bedside measures of forced vital capacity may be considered to monitor the development of progressive respiratory acidosis and type 2 respiratory failure. The earliest change noted in blood gases in a patient with incipient ventilatory failure is a fall in arterial PO_2_ [10]. The arterial PCO_2_ may initially be low due to reflex tachypnea [10].

## Conclusions

This case report illustrated a patient with myasthenia gravis who developed type 2 respiratory failure in the setting of COVID-19 infection. Type 2 respiratory failure most likely occurs due to respiratory muscle weakness and myasthenic crisis. After developing type 2 respiratory failure, our patient was considered unsuitable for mechanical ventilation, IVIG, or plasma exchange and died the next day. Diagnosis of the myasthenic crisis was not confirmed on repetitive nerve stimulation or antibody titre. Neurological examination was unremarkable on admission but was not performed when he developed new type 2 respiratory failure due to his reduced GCS score. These are the limitations of this study. The high dose of steroids was probably a risk factor for his myasthenic crisis. Although an association cannot be confirmed with this case study, further research may be indicated to evaluate the efficacy and risk of steroid use in this population.
